# Pandemic effects on health condition specific healthcare encounters in British Columbia, Canada.

**DOI:** 10.23889/ijpds.v7i3.2037

**Published:** 2022-08-25

**Authors:** Jason Flindall, Saiganesh Dhannewar, Mikhail Skrigitil, Siddharth Chadda, Samantha Magnus, Heather Richards, Lisa Corscadden

**Affiliations:** 1 Ministry of Health, British Columbia Canada

## Objectives

While overall health service use declined following the start of the pandemic, the aim of this analysis is to generate insights to inform public health priorities by identifying higher-than-expected patterns of health care service use for some health condition and population groups.

## Approach

Health care encounters for hospital, emergency department, and primary care encounters between 2011 and 2021 were categorized into condition groups according to the CIHI Population Grouping Methodology (British Columbia version). Actual health condition encounters were compared with ARIMA-based encounter forecasts to identify conditions with different-from-expected encounter rates in 2020 and 2021. For each of 225 CIHI-defined health conditions, we identified health conditions for which service use was higher-than-expected. Area-based socioeconomic status and virtual care visit data are examined to further explore conditions that continue to differ from their pre-pandemic encounter patterns.

## Results

This analysis demonstrates that some health condition groups have seen dramatic increases in service use. The three most impacted groups with higher-than-expected encounters are hypercholesterolaemia/high cholesterol [47.8% increase in average monthly encounters since 2019], emotional and behavioural disorder (w/onset generally in childhood) [+37.3%] and neurotic/anxiety/obsessive compulsive disorder [+28.0%]. Since the start of the pandemic in British Columbia, the health condition groups with both the highest volumes of services and higher than expected service use included: hypercholesterolemia & hypothyroidism, mental health conditions (eating disorder, depression, and others), hypertension and heart failure, and diabetes. Additional descriptive analysis explores potential inequities in encounters by socio-economic status and how virtual care has changed service patterns.

## Conclusion

Increased service use may reflect greater need, better access to virtual care or potential changes in diagnoses. Identifying patterns of higher-than-expected use can support program planning to address growing need in certain regions or populations. Additional exploration will be undertaken to examine lower-than-expected service use as potential unmet need.

